# Keratin-Mediated Selective Inhibition in Proliferation and Selective Apoptosis of Keloid Fibroblasts

**DOI:** 10.34133/bmr.0231

**Published:** 2025-07-22

**Authors:** Hyeon Jeong Kang, Woo Gyeong Kim, Seong Yeong An, Jae-Hyung Lee, Dong Nyoung Heo, Yu-Shik Hwang

**Affiliations:** ^1^ Department of Maxillofacial Biomedical Engineering, College of Dentistry, Kyung Hee University, Seoul 02447, Republic of Korea; ^2^ KeraMedix Inc., Seoul 02455, Republic of Korea; ^3^ Department of Oral Microbiology, College of Dentistry, Kyung Hee University, Seoul 02447, Republic of Korea.; ^4^ Biofriends Inc., Seoul 02447, Republic of Korea

## Abstract

Keloids are pathological scars characterized by excessive proliferation of fibroblasts and abnormal extracellular matrix (ECM) accumulation, largely mediated by transforming growth factor-β1 (TGF-β1). Current therapeutic approaches often fail due to high recurrence and limited selectivity. Here, we investigate the potential of human hair-derived keratin (HK) as a biomaterial with selective anti-fibrotic activity. Using multiple in vitro models including 2D monolayers, 3D spheroids, fibroblast–keratinocyte coculture, and collagen gel contraction, we evaluated the effects of 0.5% HK on keloid fibroblasts (KFs) and normal dermal fibroblasts (DFs), with and without TGF-β1 stimulation. HK selectively inhibited KF proliferation, viability, and migration while sparing DF. In 3D models, HK significantly reduced KF-mediated spheroid expansion and collagen matrix contraction, even under profibrotic stimulation. Mechanistically, HK activated intrinsic apoptotic signaling, up-regulating pro-apoptotic proteins (Bax, caspase-3, CYCS) and down-regulating Bcl-2 and XIAP. Transcriptomic profiling revealed that HK down-regulated pathways associated with ECM–receptor interaction, focal adhesion, and aminoacyl-tRNA biosynthesis in KF, suggesting a dual modulation of fibrotic remodeling and mitochondrial function. These findings demonstrate that HK exerts selective anti-fibrotic and pro-apoptotic effects on pathological fibroblasts, with minimal impact on normal cells. By modulating both ECM organization and cell survival pathways, keratin demonstrates strong potential as a therapeutic biomaterial for targeted keloid treatment.

## Introduction

Keloids are pathological scars that result from aberrant wound healing, characterized by excessive extracellular matrix (ECM) deposition and sustained fibroblast proliferation that extends beyond the original wound boundary [1–3]. Unlike hypertrophic scars, keloids rarely undergo spontaneous regression and are frequently associated with chronic pain, pruritus, and considerable psychological distress, all of which significantly impair patients’ quality of life [4]. At the molecular level, dysregulation of the transforming growth factor-β1 (TGF-β1) signaling pathway plays a central role in keloid pathogenesis by promoting fibroblast activation and abnormal collagen accumulation [1,5,6]. Up-regulation of TGF-β1 and its downstream mediators, such as tissue inhibitors of metalloproteinases (TIMPs), together with reduced activity of matrix metalloproteinases (MMPs), contributes to a persistent ECM imbalance and pathological scarring [6,7].

Although a wide range of therapeutic options have been employed including corticosteroid injections, surgical excision, radiation therapy, laser treatment, and silicone-based compression, the clinical management of keloids remains challenging due to high recurrence rates and the risk of damage to adjacent healthy tissue [2,8,9]. These limitations highlight the urgent need for biologically active and selective therapeutic strategies that can modulate fibrotic activity without disrupting normal tissue homeostasis.

HK, a structural protein abundantly expressed in hair, skin, and nails, has recently emerged as a promising biomaterial for wound healing and tissue regeneration [10]. HK is biodegradable and biocompatible and possesses favorable characteristics such as its ability to modulate oxidative stress and supporting ECM remodeling [11]. Although direct evidence of HK modulation of TGF-β1 signaling or fibrotic gene expression in keloid tissue is still lacking, previous studies in other tissue models have demonstrated its capacity to influence fibroblast behavior and mitigate fibrotic responses [12]. However, the specific effects of HK on pathological fibroblasts, particularly keloid fibroblasts (KFs), have not been fully defined. Furthermore, its potential to modulate TGF-β1-induced fibrotic activity within a 3-dimensional (3D) microenvironment has yet to be thoroughly explored.

In this study, we systematically evaluated the effects of HK on KF proliferation, viability, and fibrotic activity using multiple in vitro models, including 2D monolayer cultures, coculture systems, 3D spheroids, and collagen gel contraction assays. We further examined whether HK could modulate TGF-β1-induced fibrotic responses under both serum-rich and serum-free conditions. Collectively, our findings highlight the potential of HK as a selective anti-fibrotic biomaterial that can target pathological fibroblast activity while preserving the viability of dermal fibroblasts (DFs), supporting its potential application in the treatment of pathological scarring.

## Materials and Methods

### Cell culture

KFs were obtained from the American Type Culture Collection (Manassas, VA, USA). Human epithelial keratinocyte (HEK) and DF were purchased from CEFObio (Seoul, Korea). Fibroblast cell types were cultured in Dulbecco’s modified Eagle’s medium (DMEM; Sigma-Aldrich, St. Louis, MO, USA) supplemented with 10% (v/v) fetal bovine serum (FBS; Gibco, Grand Island, NY, USA) and 1% (v/v) penicillin–streptomycin (Gibco), whereas HEK cells were cultured in EpiLife Medium (Gibco) supplemented with Human Keratinocyte Growth Supplement (Gibco) and 1% (v/v) penicillin–streptomycin (Gibco). Cells were incubated at 37 °C in a humidified atmosphere containing 5% CO_2_, and the culture medium was replaced every 2 d. For subculturing, cells were detached using Accutase solution (Sigma-Aldrich) according to the manufacturer’s instructions. KF and DF cells within 10 passages and HEK cells within 3 passages were used for experiments.

### Preparation of HK

HK was prepared following the method described [11]. Human hair was washed using general detergent and delipidized with chloroform (Junsei Chemical):methanol (Merck Millipore) (2:1, v/v) for 24 h at room temperature. The delipidized hair was oxidized with 2% (w/v) peracetic acid (Sigma-Aldrich) for 12 h at 37 °C. The hair was reacted with Shindai solution [5 M urea (Sigma-Aldrich), 2.4 M thiourea (Sigma-Aldrich), 5% 2-mercaptoethanol (Sigma-Aldrich), 24 mM trizma base (Sigma-Aldrich), pH 8.5] for 72 h at 50 °C. After reaction, the mixture was centrifuged at 3,500 rpm for 20 min and the supernatant was dialyzed against deionized water for 5 d with 3 changes in water a day. The solution of HK was centrifuged at 3,500 rpm for 20 min, and the supernatant was lyophilized using a freezer-dryer.

### TGF-β1 treatment

To induce a fibrotic phenotype, KF and DF were seeded at a density of 5 × 10^4^ cells per well in 24-well plates and allowed to adhere overnight. Cells were then cultured in either DMEM supplemented with 10% (v/v) FBS or serum-free DMEM, according to the experimental design. Human TGF-β1 (PeproTech, USA) was added at a final concentration of 10 ng/ml. Where indicated, HK (0.5%, w/v), prepared as described in the previous section, was added concurrently with TGF-β1.

### 2D proliferation and viability assays

#### CCK-8 assay

Cell viability and proliferation were assessed using a Cell Counting Kit-8 (CCK-8; Dojindo Laboratories, Kumamoto, Japan) according to the manufacturer’s instructions. KF and DF were seeded in 96-well plates at a density of 5 × 10^3^ cells/well and treated with or without TGF-β1 and HK. At days 1, 2, 3, and 4, 10 μl of CCK-8 solution was added to each well, followed by incubation at 37 °C for 1 h. Absorbance was measured at 450 nm using a microplate spectrophotometer (Mobi; MicroDigital, Seoul, Korea).

#### Live/Dead staining

Qualitative assessment of cell viability was performed using the LIVE/DEAD Viability/Cytotoxicity Kit (Thermo Fisher Scientific). Cells were incubated with the staining solution for 30 min at room temperature in the dark. Live cells (green fluorescence) were stained with calcein-AM, and dead cells (red fluorescence) were stained with ethidium homodimer-1. Fluorescence images were captured using an inverted fluorescence microscope (IX71, Olympus, Tokyo, Japan), and at least 3 randomly selected fields were imaged per well for comparison.

### 3D keloid spheroid formation and analysis

#### Spheroid formation

To generate 3D spheroids, KFs (5 × 10^5^ cells/well) were seeded into 3D petri dish micro-mold spheroid plates (MicroTissues Inc., Providence, RI, USA). Spheroids were formed spontaneously under standard culture conditions in DMEM supplemented with 10% (v/v) FBS for 24 h.

#### Spheroid expansion under TGF-β1 and HK cotreatment

After spheroid formation, cultures were treated with or without human TGF-β1 and HK in spheroid maintenance medium. Spheroid diameters were measured at days 0, 1, 4, and 7 using ImageJ software (National Institutes of Health, Bethesda, MD, USA). Changes in spheroid size were calculated relative to baseline (day 0).

#### Long-term culture and histological/immunohistological evaluation

To generate multicellular 3D spheroids, KFs (5 × 10^5^ cells/well) were seeded into 3D petri dish micro-mold spheroid plates (MicroTissues Inc., Providence, RI, USA) for 1 d and human keratinocytes (5 × 10^5^ cells/well) were loaded on the previously formed cell spheroids within 3D petri dish micro-mold spheroid plates. The multicellular 3D spheroids were cultured for 3 d in 1:1 mixed culture medium of DMEM supplemented with 10% (v/v) FBS and EpiLife Medium (Gibco) supplemented with Human Keratinocyte Growth Supplement (Gibco) and 1% (v/v) penicillin–streptomycin (Gibco). After 3 d of culture within microwells, the multicellular spheroids were retrieved from microwell and then subsequently transferred to vessels of a high aspect ratio vessel (HARV) bioreactor (Synthecon) for long-term culture over 7 d. Each vessel containing multicellular cell spheroids was rotated anticlockwise at 30 rpm throughout the whole culture period in the presence of TGF-β1 or 0.5% HK. At the endpoint, spheroids were fixed in 4% paraformaldehyde, embedded in paraffin, and sectioned at 5-μm thickness. Sections were stained with hematoxylin and eosin (H&E) and imaged using a bright-field microscope. Histological changes were qualitatively evaluated based on cell density and the degree of stratified cell layer formation. Paraffin-embedded spheroid sections were deparaffinized and rehydrated prior to immunofluorescence staining. Primary antibodies included mouse anti-E-cadherin (Abcam, 1:1,000) and rabbit anti-vimentin (Abcam, 1:1,000), which were applied and incubated overnight at 4 °C. The following day, Alexa Fluor 488-conjugated goat anti-mouse IgG (Invitrogen, 1:500) and Alexa Fluor 594-conjugated goat anti-rabbit IgG (Invitrogen, 1:500) were applied for 1 h at room temperature. Nuclei were counterstained with 4′,6-diamidino-2-phenylindole (DAPI) (Sigma-Aldrich, USA). Fluorescence images were acquired using an inverted fluorescence microscope. Expression patterns of E-cadherin and vimentin were qualitatively compared to assess epithelial–mesenchymal transition (EMT)-related changes in response to TGF-β1 and HK treatment.

### Human apoptosis antibody array

Total cellular proteins were extracted from spheroids using the lysis buffer provided in the kit, and concentration was measured using a BCA Protein Assay Kit (Abcam). Equal amounts of protein (200 to 400 μg per membrane) were incubated with the Human Apoptosis Antibody Array (Abcam) according to the manufacturer’s instructions. Protein signals were visualized using ECL Western Blotting Substrate (Thermo Fisher Scientific) and detected on x-ray film (AGFA, Mortsel, Belgium). Signal intensities were analyzed using ImageJ software and normalized to internal controls on the membrane.

### Collagen gel contraction assay

Type I collagen derived from rat tail (Gibco) was used to prepare 3D collagen matrices at a final concentration of 2 mg/ml. The gel solution was prepared on ice by mixing the collagen stock solution, phosphate-buffered saline (PBS), 1 N NaOH, and sterile distilled water according to the manufacturer’s instructions to achieve physiological pH 7.4. The mixture of KF and DF (5 × 10^5^ cells/well) was suspended in the collagen mixture and dispensed into 24-well plates. After polymerization at 37 °C for 30 min in a humidified incubator, the gels were overlaid with culture medium containing 10% (v/v) FBS, with TGF-β1 and/or HK. Gel contraction was monitored and imaged at days 0, 1, 4, and 7. The diameter of each gel was measured using ImageJ software.

### Cell migration assay

A 2-well physical migration assay was performed using the 2-well culture insert (Ibidi, Germany) to evaluate cell distribution and migration behavior under HK treatment. KF and DF were separately seeded into each chamber of the insert at a density of 3 × 10^4^ cells/well in DMEM supplemented with 10% (v/v) FBS. After 24 h of attachment, the insert was carefully removed, leaving a cell-free gap of approximately 500 μm. Cells were then cultured under either serum-containing or serum-free conditions, with or without 0.5% HK, for up to 7 d. At days 0, 1, and 7, cell migration into the central gap was visualized by fluorescence microscopy. DF and KF were labeled with green and red fluorescent dyes, respectively, prior to seeding to distinguish the 2 populations. Cell viability and relative density within the gap were additionally assessed using Coomassie blue staining (Invitrogen).

### Whole RNA sequencing analysis

#### RNA extraction and sequencing

For the RNA sequencing analysis, total RNA was isolated from both HK-treated keloid cells and control cells (CON). The quality and integrity of the extracted RNA were evaluated using a BioAnalyzer. RNA sequencing libraries were prepared following the standard Illumina protocol (TruSeq Stranded mRNA Library Prep Kit). Approximately 300–base pair (bp) fragments were selected via gel electrophoresis and then amplified by polymerase chain reaction (PCR). Sequencing was performed on the Illumina platform using paired-end mode (2 × 101 bp).

#### RNA sequencing data processing

Sequencing adapters were removed using cutadapt (http://cutadapt.readthedocs.io/en/stable/), followed by quality assessment of raw reads. High-quality reads were aligned to the human reference genome (hg38) with HISAT2 (v2.2.1) [13]. Only uniquely and properly mapped read pairs were retained for downstream analysis. Gene annotations were acquired from Ensembl (release 98) biomart (http://www.ensembl.org/). To evaluate expression levels of genes, the TPM (transcript per million reads) measurement unit was used. Differentially expressed genes (DEGs) between HK-treated and CON cells were identified using Fisher’s exact test, with significance thresholds set at ≥2-fold expression change, ≥5 TPM expression, and 5% false discovery rate (FDR). Protein functional classification of DEGs was performed using PANTHER (Protein ANalysis THrough Evolutionary Relationships) tools (http://www.pantherdb.org/). Gene ontology (GO) term and Kyoto Encyclopedia of Genes and Genomes (KEGG) pathway enrichment analysis were performed using DAVID (The Database for Annotation, Visualization and Integrated Discovery) functional annotation tools [14].

### Statistical analysis

All quantitative data are presented as mean ± standard deviation (SD) from at least 3 independent experiments. Image analysis was performed using ImageJ software. Statistical calculations were conducted using Microsoft Excel. Two-way analysis of variance (ANOVA) was used to evaluate the effects of treatment conditions and time points, followed by Tukey’s post hoc test for multiple comparisons. A *P* value of <0.05 was considered statistically significant.

## Results

### HK selectively inhibits the proliferation of KFs

**Fig. 1.**
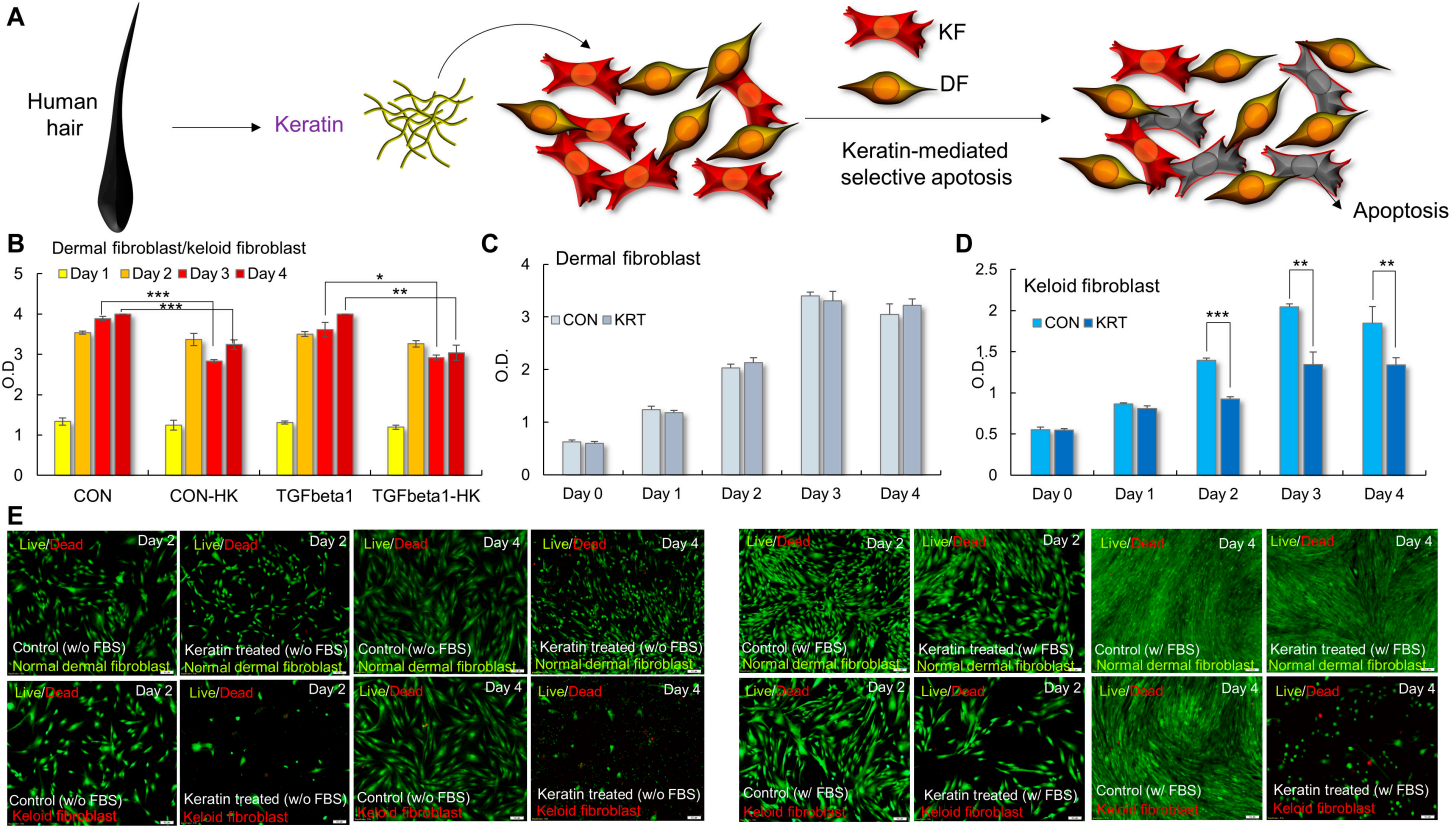
Schematic and experimental validation of HK-mediated selective suppression of KF versus DF. (A) Schematic illustration of HK inducing selective apoptosis in KF, with minimal impact on DF. The schematic summarizes the HK-induced differential response, where KF undergoes apoptosis upon HK treatment. (B) OD values from the CCK-8 assay measured over 4 d in DF and KF treated with HK (CON-HK), TGF-β1, or TGF-β1 + HK. HK significantly suppressed KF proliferation from day 2 onward. (C) DF proliferation was unaffected by HK treatment. (D) HK-treated KFs exhibited statistically significant reduction in proliferation from day 2 compared to CON. (E) Representative Live/Dead fluorescence images of DF and KF at days 2 and 4, under serum-containing or serum-free conditions with or without 0.5% HK. Live cells are stained green, and dead cells are red. KFs showed increased cell death, especially under serum-free conditions. Data are presented as mean ± SD (*n* = 3). Statistical significance was determined using 2-way ANOVA followed by Tukey’s post hoc test (*P < 0.05, **P < 0.01, ***P < 0.001).

To investigate the selective anti-fibrotic effects of HK-treated HEK, we evaluated both the proliferation and viability of DF and KF through CCK-8 assays and Live/Dead staining over 4 d (Fig. 1). In CCK-8 assays, untreated KF showed continuous proliferation over the 4-d period, whereas HK-treated KF (CON-HK) exhibited a significant reduction in optical density (OD) from day 2 onward (Fig. 1B). This inhibitory effect was further enhanced by cotreatment with TGF-β1 (TGF-β1 + HK), compared to TGF-β1 alone. In contrast, HK treatment had no significant impact on DF proliferation. As shown in Fig. 1C, HK-treated DFs maintained OD values comparable to CON across all time points, indicating minimal cytotoxicity. In KFs, however, a statistically significant suppression of proliferation was observed in the HK-treated group starting at day 2 and became more pronounced by days 3 and 4 (*P* < 0.05; Fig. 1D).

To evaluate the viability effects of HK beyond proliferation, Live/Dead staining was performed on DF and KF cultures at days 2 and 4, with or without HK, under serum-containing (w/ FBS) and serum-free (w/o FBS) conditions (Fig. 1E). In DF cultures, the majority of cells remained viable (green) regardless of HK treatment or serum conditions, indicating that 0.5% HK did not compromise DF survival. In contrast, a distinct increase in dead cells (red fluorescence) was observed in KFs following HK treatment, particularly under serum-free conditions. On day 4, HK-KFs showed a substantial loss of viability compared to CON, while under serum-rich conditions the effect was moderate but still apparent. These results demonstrate that HK selectively impairs KF viability, with cytotoxicity becoming more pronounced in serum-free FBS conditions.

Collectively, these results demonstrate that HK selectively suppresses proliferation and induces cell death in KFs while sparing normal DFs. This dual effect was more prominent under stress conditions such as serum deprivation, suggesting that HK mediates context-dependent apoptosis in KF.

### HK induces intrinsic apoptosis in KF spheroids under serum-free conditions

**Fig. 2.**
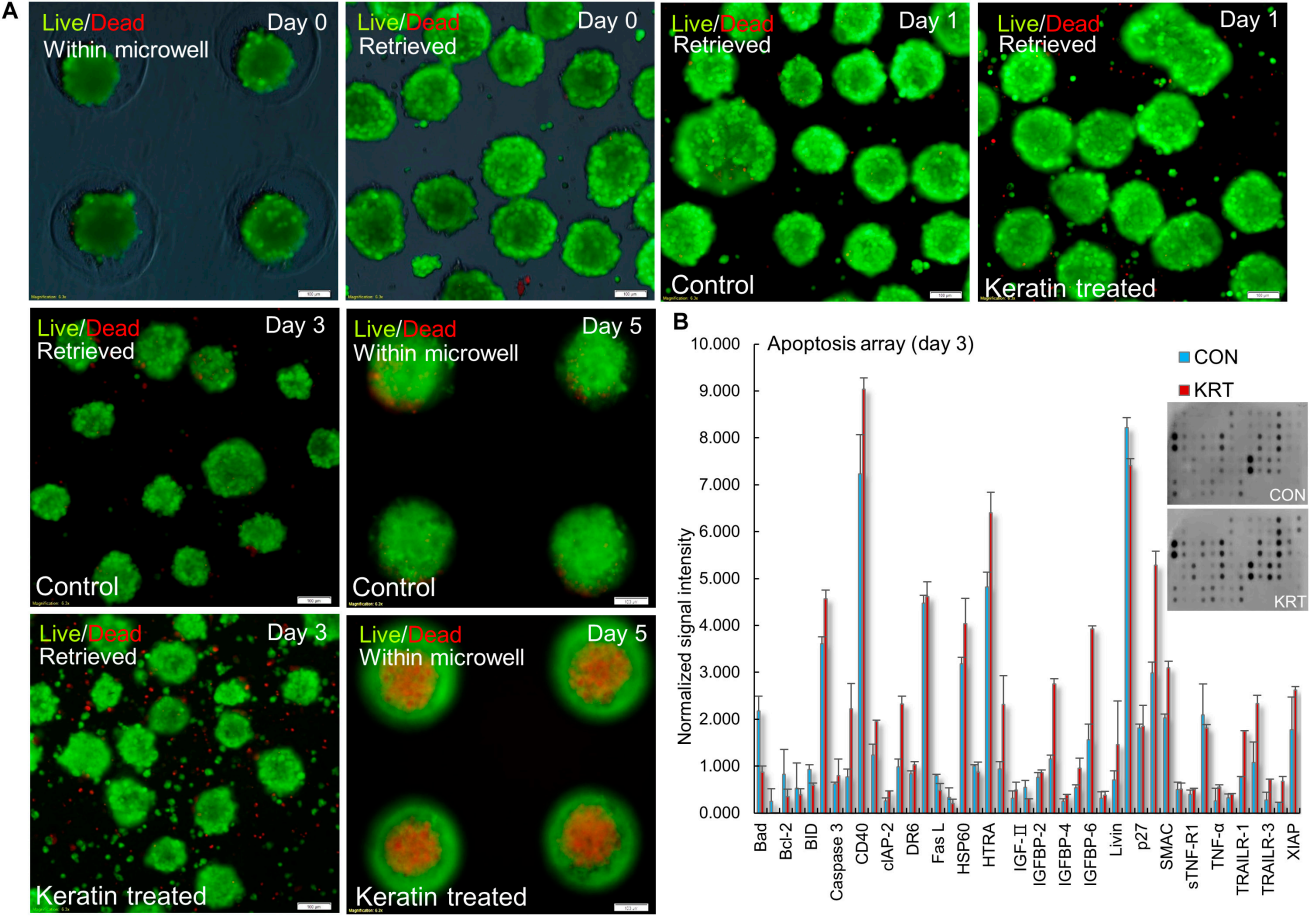
Live/Dead stage of KF spheroids and apoptosis array. (A) Representative fluorescence images of KF spheroids cultured under serum-free conditions. Spheroids were formed in microwell arrays and retrieved at days 0, 1, 3, and 5. CON and HK groups were compared. Live cells are shown in green and dead cells in red. Minimal cell death was observed at day 0. HK-treated spheroids showed a marked increase in dead cells by day 3, which became more prominent by day 5, whereas CON spheroids maintained viability and compact morphology throughout. (B) Quantification of an apoptosis antibody array performed at day 3. HK spheroids exhibited up-regulation of pro-apoptotic markers and down-regulation of anti-apoptotic proteins compared to CON. Notably, CYCS and HTRA2 showed up-regulation, suggesting that HK activates the intrinsic apoptotic pathway via mitochondrial signaling. Right: Representative dot blot images (top, CON; bottom, HK). Data are presented as mean ± SD and normalized to internal CON.

To investigate the pro-apoptotic effects of HK in a 3D environment, KFs were cultured in microwell arrays under serum-free conditions to form spheroids, and Live/Dead staining was performed at multiple time points (Fig. 2A). At day 0, both CON and HK groups formed compact, viable spheroids with minimal cell death. However, by day 3, HK spheroids exhibited a noticeable increase in red fluorescence, indicative of apoptotic cell death, which became more prominent by day 5. It is recognized that large spheroids can develop core necrosis or apoptosis due to limited oxygen and nutrient diffusion [15,16]. However, the more pronounced and spatially extended cell death observed in the HK spheroids suggests an additional pro-apoptotic effect of HK, beyond the nature core cell death commonly associated with 3D culture. In contrast, CON spheroids retained viability and structural integrity over the same period. To further elucidate the mechanism of cell death, an apoptosis antibody array was performed at day 3 (Fig. 2B). HK spheroids showed elevated expression of pro-apoptotic markers associated with both intrinsic and extrinsic pathways, including Bax, caspase-3, cytochrome c (CYCS), death receptor 6 (DR6), Fas ligand (FasL), and high temperature requirement A2 (HTRA2) [17,18]. Conversely, anti-apoptotic proteins such as Bcl-2 and XIAP were significantly down-regulated in the HK group. Notably, CYCS and HTRA2 were among the strongly up-regulated proteins, suggesting that HK triggers mitochondrial membrane permeabilization, thereby activating the intrinsic apoptotic cascade [19]. Additionally, increased expression of ECM and cytoskeletal genes was observed in certain HK spheroid regions, potentially reflecting stress-induced remodeling or compensatory adaptation to HK exposure. In conclusion, this study demonstrates that HK effectively induces intrinsic apoptosis in KF under serum-free 3D culture conditions. These findings provide a foundation for exploring HK-based strategies in managing pathological fibroblast proliferation, particularly in KF.

### HK inhibits TGF-β1-induced keloid spheroid expansion and regulates gene expression in KFs

**Fig. 3.**
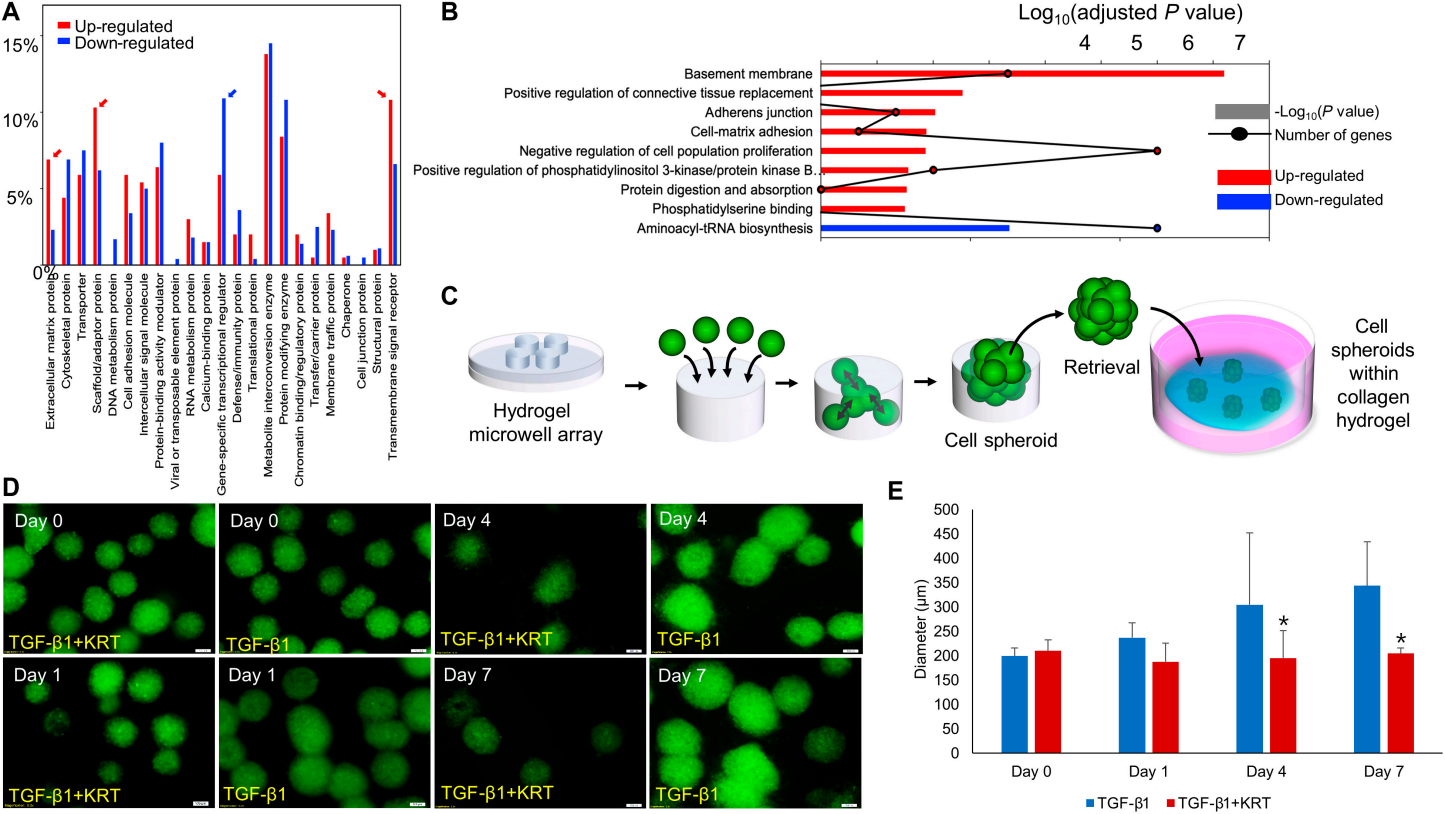
HK suppresses TGF-β1-induced spheroid expansion and remodels gene expressions in KFs. (A) DEGs classified by PANTHER protein class system. Up-regulated DEGs were enriched in ECM- and signaling-related proteins. (B) GO/KEGG pathway analysis showing that fibrotic remodeling pathways were up-regulated, while metabolic and translational pathways were suppressed. (C) Schematic of the microwell-based spheroid formation and collagen gel embedding. (D) Representative fluorescence images of spheroids over 7 d under TGF-β1 or TGF-β1 + HK conditions. (E) Quantification of spheroid diameter showing significantly reduced growth in the HK-treated group from day 4 onward. Data are presented as mean ± SD (*n* = 3).

To investigate the long-term effect of HK on KF remodeling and metabolism under fibrotic stimulation, we employed a 2-part approach combining transcriptomic profiling and 3D spheroid modeling (Fig. 3). RNA sequencing of HK-treated and untreated KF revealed a total of 2,004 differentially expressed genes (DEGs), including 334 up-regulated and 1,670 down-regulated transcripts, at thresholds of |fold change| ≥ 2, TPM ≥ 5, and FDR < 5%. Functional categorization using the PANTHER protein class system showed that ECM protein, scaffold/adaptor protein, and transmembrane signal receptor categories were highly enriched among the up-regulated DEGs (Fig. 3A). Furthermore, GO and KEGG analyses identified significant enrichment of pathways related to tissue architecture, including basement membrane organization, adherens junction, and cell–matrix adhesion. Conversely, pathways related to protein translation and mitochondrial function, such as aminoacyl-tRNA biosynthesis and phosphatidylserine binding, were notably down-regulated (Fig. 3B). These transcriptomic results suggest that HK modulates both extracellular and intracellular pathways associated with fibrosis and energy metabolism [20].

To functionally validate these findings, we utilized a hydrogel-based microwell array to generate uniform KF spheroids, which were then embedded within collagen I hydrogels and cultured under TGF-β1 or TGF-β1 + HK conditions (Fig. 3C). Time-lapse imaging from day 0 to day 7 showed that spheroids in the TGF-β1 group progressively expanded, whereas those treated with HK maintained a compact morphology with limited growth (Fig. 3D). Quantitative image analysis confirmed a significant reduction in spheroid diameter in the TGF-β1 + HK group starting at day 4 and continuing through day 7 (Fig. 3E). Moreover, fluorescence signal intensity, used as a surrogate for cellular activity, declined more rapidly in the HK-treated spheroids, indicating reduced viability or proliferation. Together, these results demonstrate that HK effectively inhibits TGF-β1-induced fibrotic expansion of KFs in a 3D matrix and simultaneously alters gene expression programs associated with ECM remodeling and mitochondrial metabolism. These findings support a dual-action model in which HK impairs both structural and metabolic drivers of pathological fibrosis.

### HK preferentially reduces KF density in coculture systems under both serum-rich and serum-free conditions

**Fig. 4.**
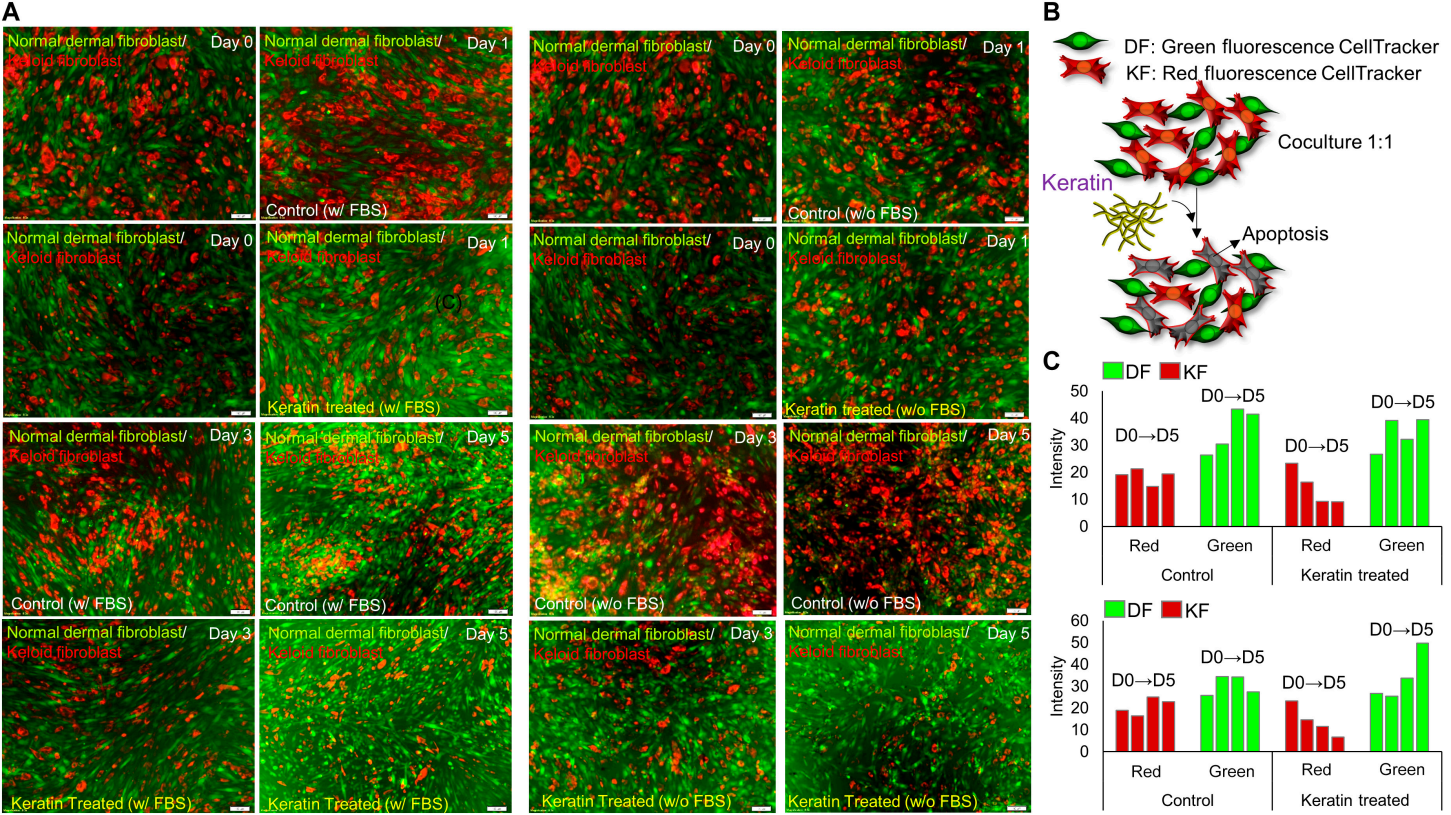
HK selectively reduces KF density in coculture systems. (A) Fluorescence images of DF (green) and KF (red) cocultures under serum-rich (w/ FBS) and serum-free (w/o FBS) conditions, from day to day 5, with or without HK treatment. (B) Schematic illustration of DF/KF coculture setup with HK-induced selective apoptosis. (C) Quantitative analysis of red and green fluorescent signal (KF and DF) area expressed as a percentage of total image area across conditions and time points.

To investigate the selective cytotoxicity of HK in a mixed-cell environment, DF and KF were cocultured and differentially labeled with green and red CellTracker dyes, respectively. Cultures were maintained in either serum-rich or serum-free conditions and monitored over 5 d to assess changes in cell distribution and relative abundance (Fig. 4A). A schematic of the coculture system and HK-induced selective apoptosis is presented (Fig. 4B). In serum-rich conditions, KF typically exhibits robust proliferation, which can highlight the apoptotic effect of HK more clearly. However, on days 3 and 5, the proportion of red-labeled KF gradually decreased in the HK group, while green-labeled DF remained predominant. In contrast, under serum-free conditions, the overall proliferation rate of both cell types is lower due to nutrient deprivation, yet HK-treated KF appeared more vulnerable under these metabolically stressed settings. While both cell types showed mild reductions in density due to nutrient deprivation, HK-treated cultures displayed a substantial loss of red fluorescence over time, indicating that HK exacerbated KF vulnerability under metabolic stress. In contrast, DF populations remained relatively stable (Fig. 4A). To quantitatively assess this trend, the fluorescent area of red and green signals was measured as a percentage of total image area (Fig. 4C). The analysis revealed a progressive reduction in red fluorescence over time in the HK group, particularly under serum-free conditions, whereas green signal intensity remained relatively stable across conditions. These findings suggest that FBS supplementation generally promotes KF proliferation, making HK selective cytotoxicity more apparent, while serum-free conditions impose additional metabolic stress that further enhances KF apoptosis. Collectively, these results support the conclusion that HK preferentially suppresses KF in coculture model, indicating its potential utility under both serum-rich and serum-free conditions.

### HK selectively inhibits spatial expansion of KFs in a physically separated coculture system

**Fig. 5.**
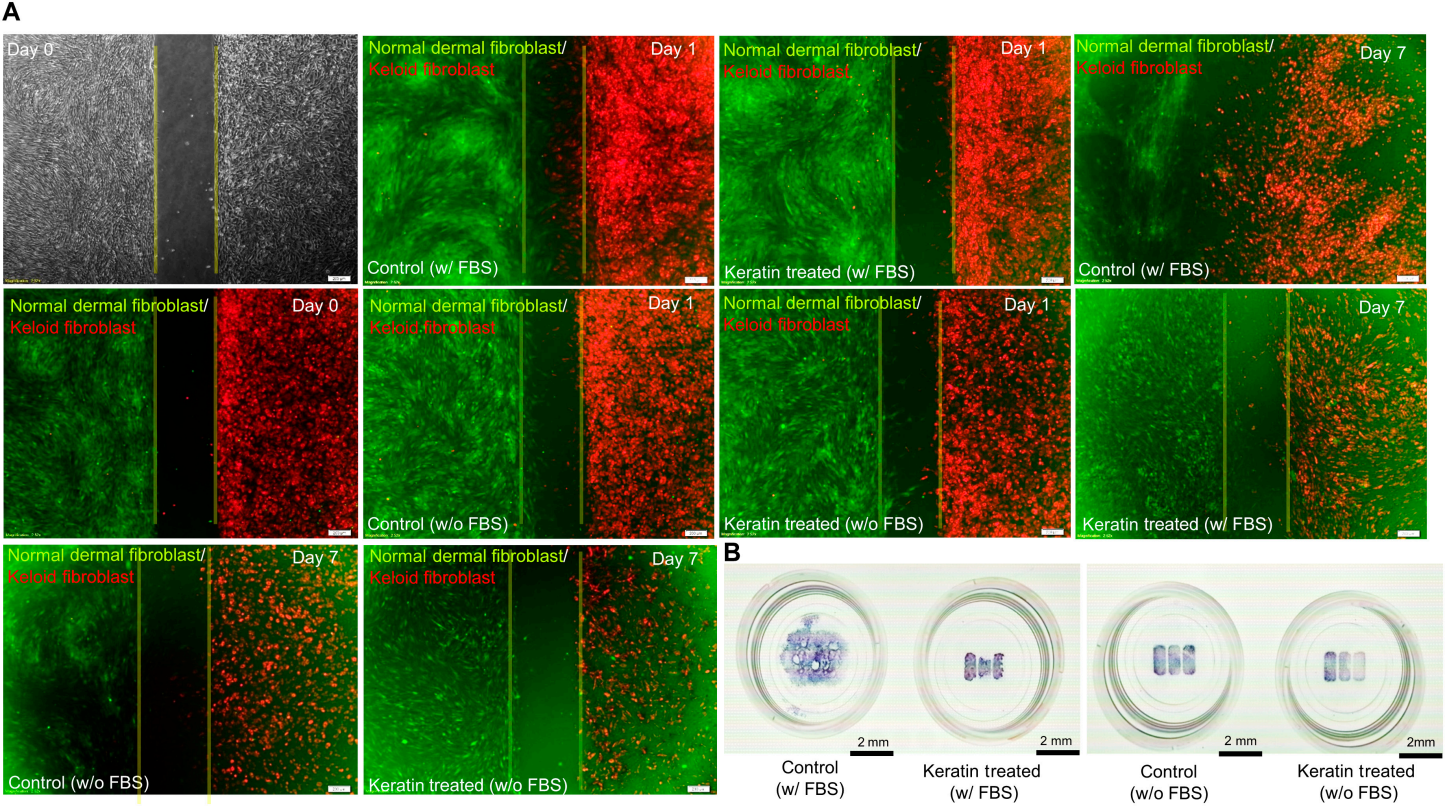
Migration assay. (A) Representative fluorescence images of physically separated cocultures of DF and KF using a 2-well insert system. Cells were seeded in opposing chambers and cultured under w/ FBS or w/o FBS conditions, with or without 0.5% HK. Images were acquired on days 0, 1, and 7. In CON groups, KF progressively migrated across the central gap, particularly under serum-rich conditions. HK treatment selectively suppressed KF migration, while DF remained largely unaffected across all conditions. (B) Bright-field images of cultures fixed and stained on day 7. The 2-well culture insert used has an internal well diameter of 6.5 mm, and the black scale bar in the figure corresponds to 2 mm in length. Residual cell density was assessed. HK-treated wells showed visibly reduced KF accumulation compared to CON, consistent with impaired pathological fibroblast expansion.

To evaluate the differential migratory behavior of DF and KF in response to HK, a physically partitioned coculture system was established using a 2-well culture insert. DF and KF were prelabeled with CellTracker green and red dyes, respectively, and seeded in separate wells. After insert removal on day 0, cells were cultured for 7 d under serum-containing or serum-free conditions with or without HK (Fig. 5A). Under serum-containing conditions, both DF and KF showed directional migration toward the center, and by day 7, KF exhibited substantial expansion beyond the initial boundary. In contrast, in serum-free conditions, the overall migration of both cell types was reduced due to the lack of mitogenic support, and KF showed minimal spread from its original position. This highlights the supportive role of FBS in maintaining cell motility and survival. HK treatment led to an additional and selective inhibition of KF expansion across both serum conditions. In the presence of FBS, HK reduced the spread of KF compared to CON. Notably, under serum-free conditions, this inhibitory effect was further enhanced, with KF confined to its original zone throughout the 7-d period. In contrast, DF exhibited stable migration and survival across all conditions. These findings suggest that HK selectively inhibits expansion pathological fibroblasts, particularly under metabolically stressed conditions. To visualize the remaining cell clusters at the endpoint, samples were fixed and stained on day 7 (Fig. 5B). It served as a proxy for cell mass and density. Consistent with fluorescence observations, HK-treated cultures showed a marked reduction in blue-stained KF clusters compared to CON.

### HK attenuates TGF-β1-induced fibroblast contractility and 3D fibrotic processing

**Fig. 6.**
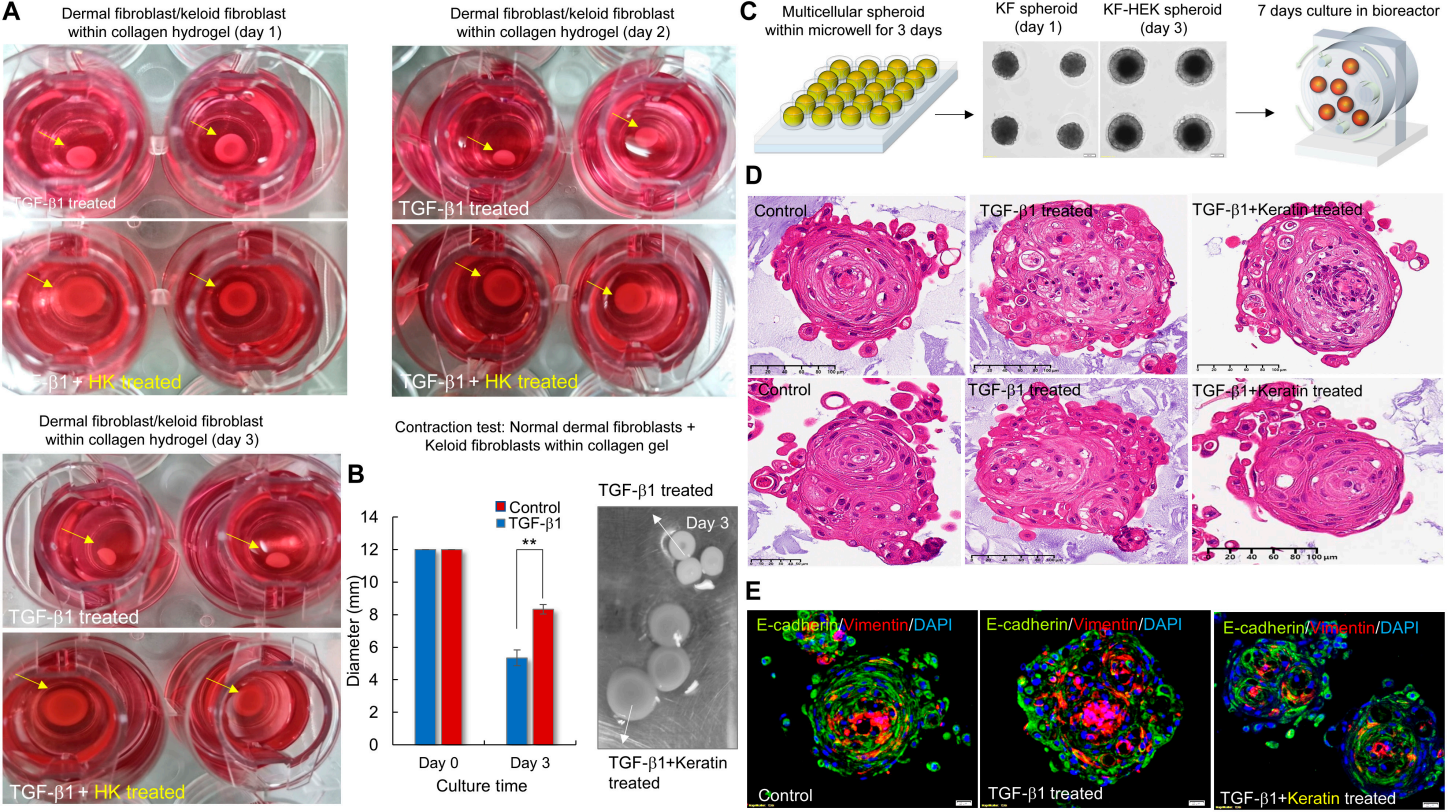
HK attenuates TGF-β1-induced gel contraction and spheroid remodeling in 3D fibrotic coculture models. (A) Representative images of collagen I hydrogels containing coencapsulated DF and KF under fibrotic stimulation. Gels were treated with TGF-β1 alone or TGF-β1 in combination with HK treatment and observed at days 1, 2, and 3. In the TGF-β1 group, visible gel contraction was observed by day 3. In contrast, cotreatment with HK resulted in visibly larger gel diameters, indicating reduced contractile activity. (B) Quantification of gel diameters measured at days 0 and 3. Gels treated with TGF-β1 + HK maintained significantly greater diameters compared to TGF-β1 alone. Right panel: Representative bright-field images of the gel surface at day 3 support the measured reduction in contraction with HK treatment. Data are presented as mean ± SD (*n* = 3). Statistical significance was determined by 2-way ANOVA with Tukey’s post hoc test. (C) Schematic of 3D spheroid culture: DF and KF were mixed and seeded into microwells to form spheroids and then transferred to a rotary bioreactor for 7-d long-term culture under fibrotic conditions. (D) H&E staining of spheroids harvested at day 7. TGF-β1-treated spheroids showed dense, multilayered cell clustering, while cotreatment with HK resulted in more uniform and less compact cell distribution. (E) Immunohistochemical staining of E-cadherin and vimentin. In the TGF-β1 group, vimentin expression was up-regulated, while E-cadherin expression was suppressed, indicating EMT induction. HK cotreatment preserved E-cadherin expression and attenuated vimentin up-regulation, suggesting inhibition of TGF-β1-induced EMT. Data are presented as mean ± SD (*n* = 3). Statistical significance was determined using 2-way ANOVA followed by Tukey’s post hoc test (**P < 0.01).

To evaluate whether HK modulates the gel contraction of fibroblasts under fibrotic stimulation, DF and KF were encapsulated in collagen I hydrogels and cultured with or without TGF-β1 and HK for up to 3 d (Fig. 6A). Visual inspection revealed progressive gel contraction in the TGF-β1 group, with a marked decrease in gel diameter by day 3. In contrast, gels treated with both TGF-β1 and HK maintained a significantly larger area, suggesting reduced contractile activity. Quantitative analysis confirmed this observation. The TGF-β1 group exhibited significant gel contraction over time, whereas cotreatment with HK attenuated this contraction effect (Fig. 6B). Bright-field images of the gels further supported these findings, showing that the diameter of the TGF-β1 + HK group remained significantly wider than that of the TGF-β1 group at day 3. To further investigate the effect of HK on multicellular keloid-like tissues for long-term culture under a 3D environment, KFs were cocultured with HEK to form spheroids using a microwell system. The spheroids were then cultured for 7 d in a bioreactor to promote long-term 3D organization (Fig. 6C). Histological analysis with H&E staining revealed that TGF-β1-treated spheroids exhibited less evident multilayer and also less faint cell junctions of peripheral region of cell spheroids. In contrast, the TGF-β1 + HK group showed a more uniform distribution of cells with well-formed cell junctions between keratinocytes (Fig. 6D). Immunofluorescence staining was further performed to assess the expression of epithelial and mesenchymal markers. In the TGF-β1-treated group, the mesenchymal marker vimentin was strongly expressed. However, in the TGF-β1 + HK group, E-cadherin expression was relatively preserved, suggesting the suppression of EMT [17] (Fig. 6E). These findings demonstrate that HK attenuates TGF-β1-induced fibroblast contractility and keloid processing in 3D cultures, highlighting its potential role in regulating tissue tension and fibrosis progression.

## Discussion

This study demonstrated that HK exerts cell type-specific effects in 2D cultures, where it selectively suppresses KF while having minimal impact on DF. In 2D monolayer assays, HK-treated KF showed significantly reduced proliferation and viability compared to CON, whereas DF maintained stable growth and viability under identical treatment conditions. These findings highlight the selective anti-fibrotic potential of HK, sparing normal cells while targeting pathological fibroblasts. Building on these 2D observations, we further investigated HK effect in a 3D culture. Unlike traditional 2D monolayers, 3D spheroids enable spatial cell–cell and cell–matrix interactions that are critical for ECM-driven remodeling and drug responsiveness, thereby providing a more tissue-relevant geometric context. Recent studies have emphasized the importance of 3D keloid models in mimicking in vivo tissue architecture and capturing clinically relevant drug responses [18,21]. In line with this, we generated keloid spheroids using a microwell-based system and analyzed their behavior under fibrotic stimulation. This approach allowed us to assess HK effects on multicellular organization, structural remodeling, and viability within a more physiologically relevant microenvironment. This 3D keloid model better reflects the dense stromal architecture of keloid lesions, including gradients of oxygens, nutrients, and local signaling, which cannot be captured in traditional monolayer cultures [21].

Mechanistically, HK induced intrinsic apoptosis in KF, with up-regulation of Bax, caspase-3, and CYCS, and down-regulation of Bcl-2 and XIAP. These findings indicate activation of mitochondrial-mediated apoptotic pathways [22–24]. Altered expression of ECM and cytoskeletal genes in 3D culture further suggests that HK may induce stress-related or compensatory structural remodeling [25]. These apoptotic changes contribute to the structural disintegration of spheroids, leading to downstream reduction in ECM production and contractile function. This mechanistic axis is supported by our observation of diminished gel contraction in HK-treated fibroblast cultures. In keloid pathophysiology, ECM remodeling is a defining feature driven by excessive collagen deposition, aberrant fibroblast matrix interactions, and impaired matrix turnover [3,6,7]. The suppression of ECM organization and cytoskeletal-related genes in HK-treated spheroids suggests that HK inhibits structural and metabolic drivers of fibrosis by disrupting the pathological feedback loop between KF and the surrounding matrix.

This antifibrotic effect was further supported by collagen gel contraction assays, where fibroblasts (DF and KF) encapsulated in collagen matrix showed TGF-β1-induced contraction, a commonly used functional indicator for fibroblast-mediated matrix remodeling and contraction activation [26]. HK treatment markedly attenuated this contraction, indicating its ability to suppress fibroblast contractility under fibrotic stimulation. In addition, a coculture system using physically separated DF and KF highlighted HK selective action. While KF exhibited aggressive migration and spatial expansion across the central gap, particularly under serum-rich conditions, HK treatment effectively restricted this behavior without compromising DF viability or movement. This spatially defined coculture system mimics tissue spatial separation and allows differential assessment of cell migration and selective cytotoxicity in a controlled interface, which better reflects in vivo fibroblast dynamics during scar formation. Furthermore, our findings indicate that FBS supplementation promotes the proliferation of KF, thereby enhancing the apoptotic effect of HK, while serum-free conditions impose additional metabolic stress that may further sensitize KF to HK-induced apoptosis. These results support the robustness of HK selective action across varying nutritional environments. Importantly, the dual selectivity of HK sparing normal fibroblasts while targeting fibrotic ones addresses a major limitation in current antifibrotic therapies that often lack cell type specificity [27,28].

Transcriptomic analysis supported these observations. RNA sequencing revealed down-regulation of pathways involved in ECM organizations, cell adhesion, migration, and proliferation. Notably, mitochondrial-related processes such as aminoacyl-tRNA biosynthesis and tRNA aminoacylation were significantly suppressed, aligning with previous findings of reduced mitochondrially encoded tRNA expression in keloid tissue [29]. These results suggest that HK may act not only as a fibrotic modulator but also as a metabolic regulator targeting bioenergetic vulnerabilities in pathological fibroblasts [30,31].

Together, these data support the therapeutic potential of HK as a dual-function anti-fibrotic agent that modulates both structural and metabolic characteristics of KF while preserving DF. Its selective action underscores its applicability in locally targeted strategies for pathological scar modulation. However, this study was limited to in vitro models. Future studies using ex vivo human skin or keloid xenografts are needed to validate HK efficacy in more physiologically relevant contexts. Further, standardization of HK processing, safety evaluation, and comprehensive mechanistic studies involving TGF-β1 signaling and mitochondrial function will be critical for clinical translation.

## Data Availability

All data including the raw RNA sequencing data that support the findings of this study are available from the corresponding author upon reasonable request.
